# Dataset on the impact of UV, nitric acid and surfactant treatments on low-density polyethylene biodegradation

**DOI:** 10.1016/j.dib.2017.07.073

**Published:** 2017-07-29

**Authors:** Vinay Mohan Pathak, Navneet Kumar

**Affiliations:** Department of Botany & Microbiology, Gurukul Kangri University, Haridwar 249404, Uttarakhand, India

**Keywords:** Biodegradation, Polymer, UV, Nitric acid, Surfactant

## Abstract

Present investigation evaluates the LDPE (low-density polyethylene) biodegradation efficiency of polymer degrading bacteria along with UV, nitric acid and surfactant treatments. In current scenario LDPE contamination reported as dominant pollutant in terrestrial and aquatic ecosystem due to its expulsion from commercial and domestic practices. Biodegradation serve as an innovative and effective approach to waste management as compared to land filling and burning processes. The outcomes of UV, nitric acid and surfactant treatments on polymer degradation in addition to bacterial treatment were determined by SEM, FT-IR and electrical conductivity analysis.

**Specifications Table**

**Table:** 

Subject area	Microbiology, Ecology, Biodegradation
More specific subject area	Outcomes of UV, nitric acid and surfactant treatments on biodegradability of low-density polyethylene samples
Type of data	Tables, Figures, Text file
How data was acquired	Exploitation of UV, nitric acid and surfactant treatments along with bacterial strains;
	SEM, FT-IR and electrical conductivity of polymer film was analyzed;
Data format	Analyzed
Experimental factors	Role of physical and chemical treatments on LDPE biodegradation
Experimental features	The relationship between the physical, chemical and biological treatments
Data accessibility	The data are available with this article

**Value of the data**

• This data could be used as systematic tool for increasing polymer degradation.
• This data will also help in developing the specific and appropriate approach for polymer degradation in a sustainable manner.
• This data represented the impact of physical and chemical treatments on the LDPE biodegradation.

## 1 Data

**Fig. 1:**
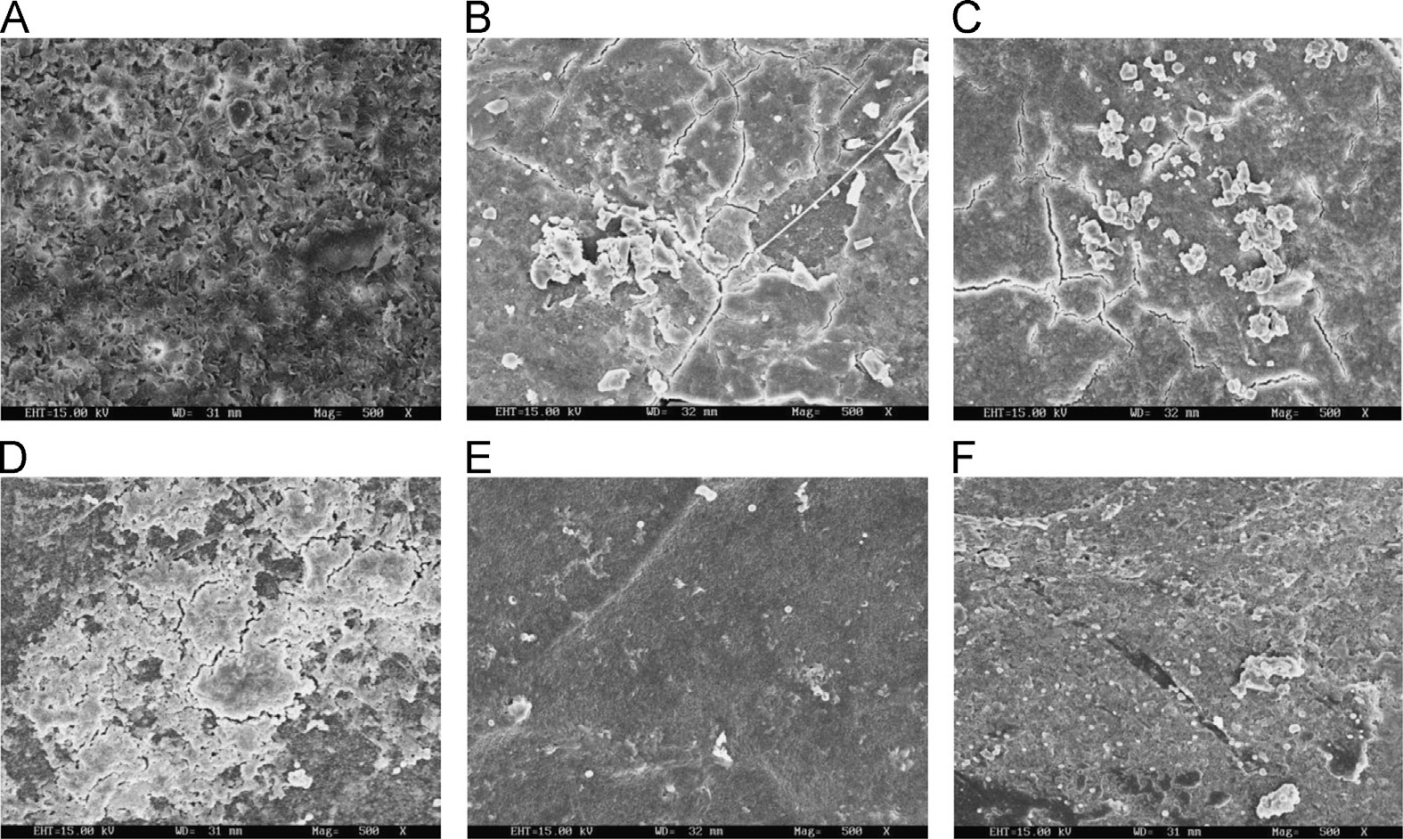
Scanning electron microscopy (SEM) micrographs of UV treated biodegraded polymer samples A- control (without UV treatment), B- *Bacillus subtilis* V8+UV treated polymer, C- *Paracoccus aminophilus* B1 4-+UV treated polymer, D- *Pseudomonas putida* C 2 5+UV treated polymer, E- *Pseudomonas aeruginosa* V1+UV treated polymer, F- *Acinetobacter calcoaceticus* V4+UV treated polymer after incubation.

**Fig. 2:**
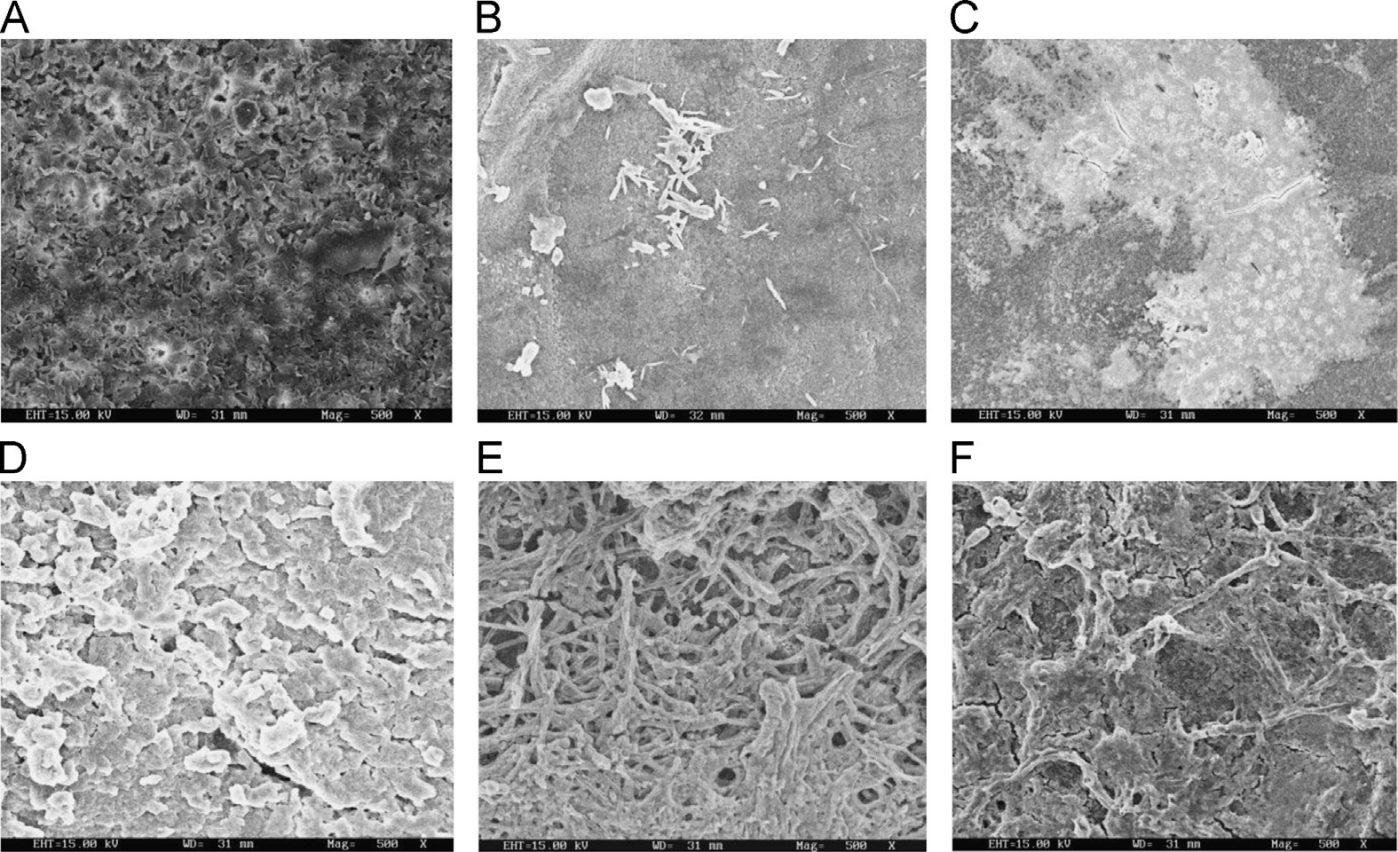
Scanning electron microscopy (SEM) micrographs of nitric acid treated biodegraded polymer samples A- control (without nitric acid treatment), B- *Bacillus subtilis* V8+ nitric acid treated polymer, C-*Paracoccus aminophilus* B1 4-+ nitric acid treated polymer, D-*Pseudomonas putida* C 2 5+ nitric acid treated polymer, E-*Pseudomonas aeruginosa* V1+ nitric acid treated polymer, F- *Acinetobacter calcoaceticus* V4+ nitric acid treated polymer after incubation.

**Fig. 3:**
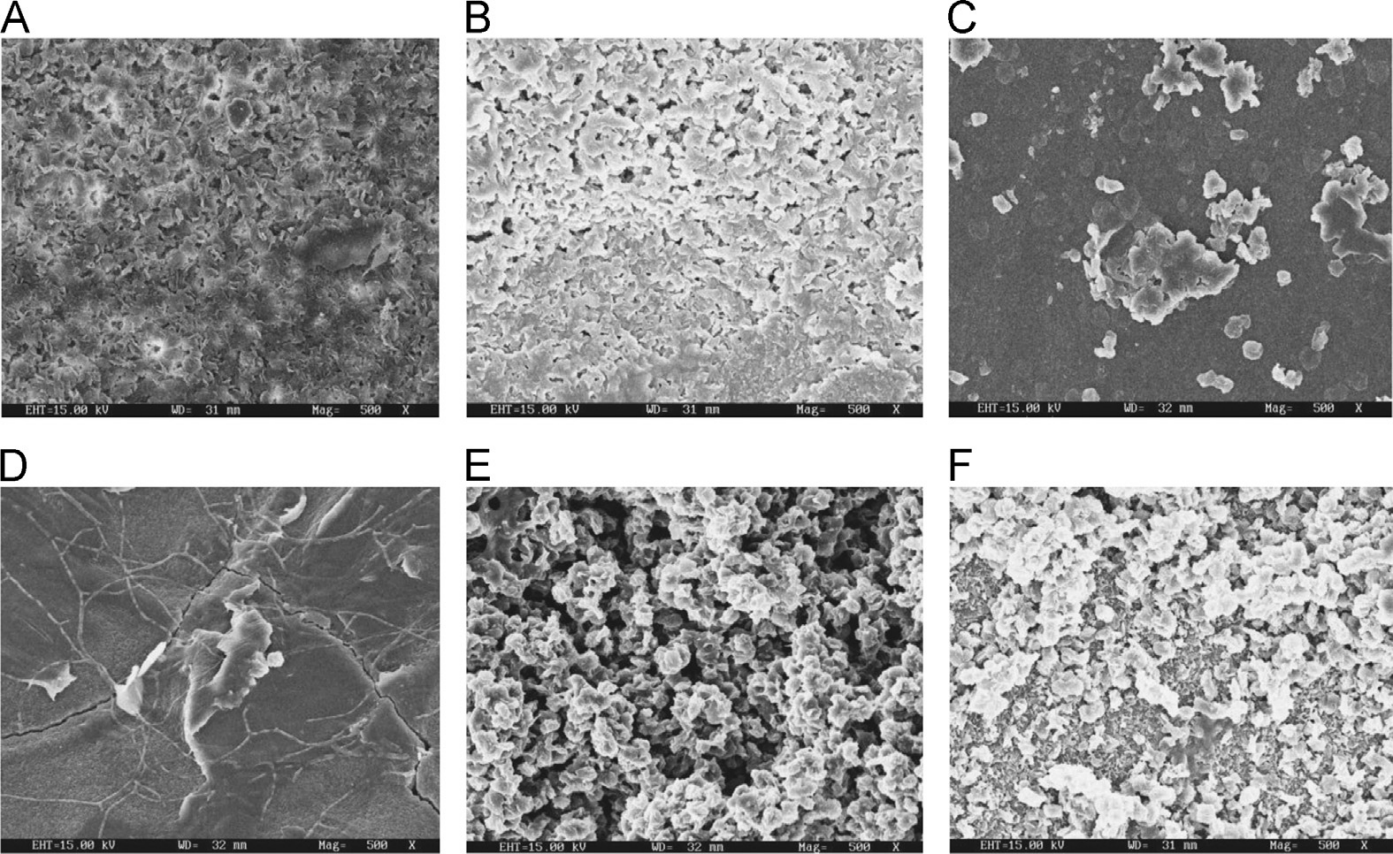
Scanning electron microscopy (SEM) micrographs of surfactant treated biodegraded polymer samples A- control (without surfactant treatment), B- *Bacillus subtilis* V8+ surfactant treated polymer, C- *Paracoccus aminophilus* B1 4-+ surfactant treated polymer, D- *Pseudomonas putida* C 2 5+ surfactant treated polymer, E- *Pseudomonas aeruginosa* V1+ surfactant treated polymer, F- *Acinetobacter calcoaceticus* V4+ surfactant treated polymer after incubation.

**Fig. 4:**
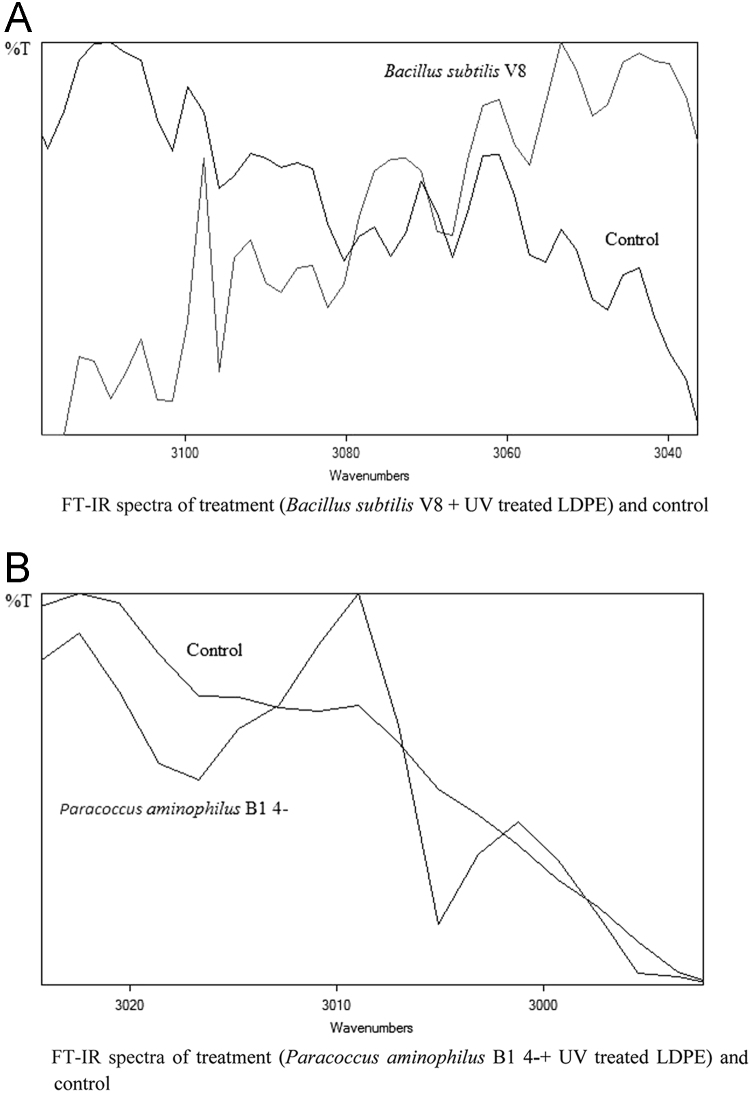

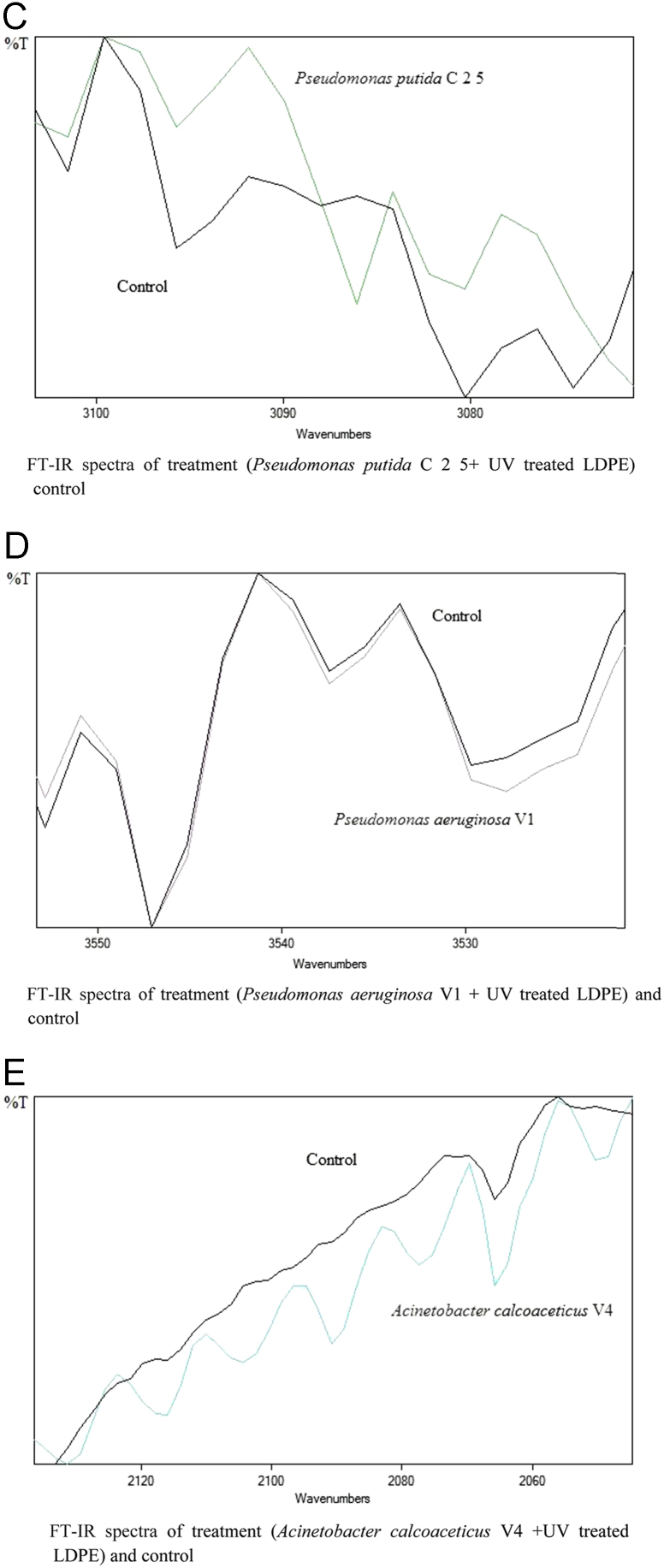
Graph A-E for bacterial treatments with control (untreated LDPE). FT-IR spectra in A for treatment (*Bacillus subtilis* V8 + UV treated LDPE), B for treatment (*Paracoccus aminophilus* B1 4- + UV treated LDPE), C for treatment (*Pseudomonas putida* C 2 5 + UV treated LDPE), D for treatment (*Pseudomonas aeruginosa* V1 + UV treated LDPE) and E for treatment (*Acinetobacter calcoaceticus* V4 + UV treated LDPE).

**Fig. 5:**
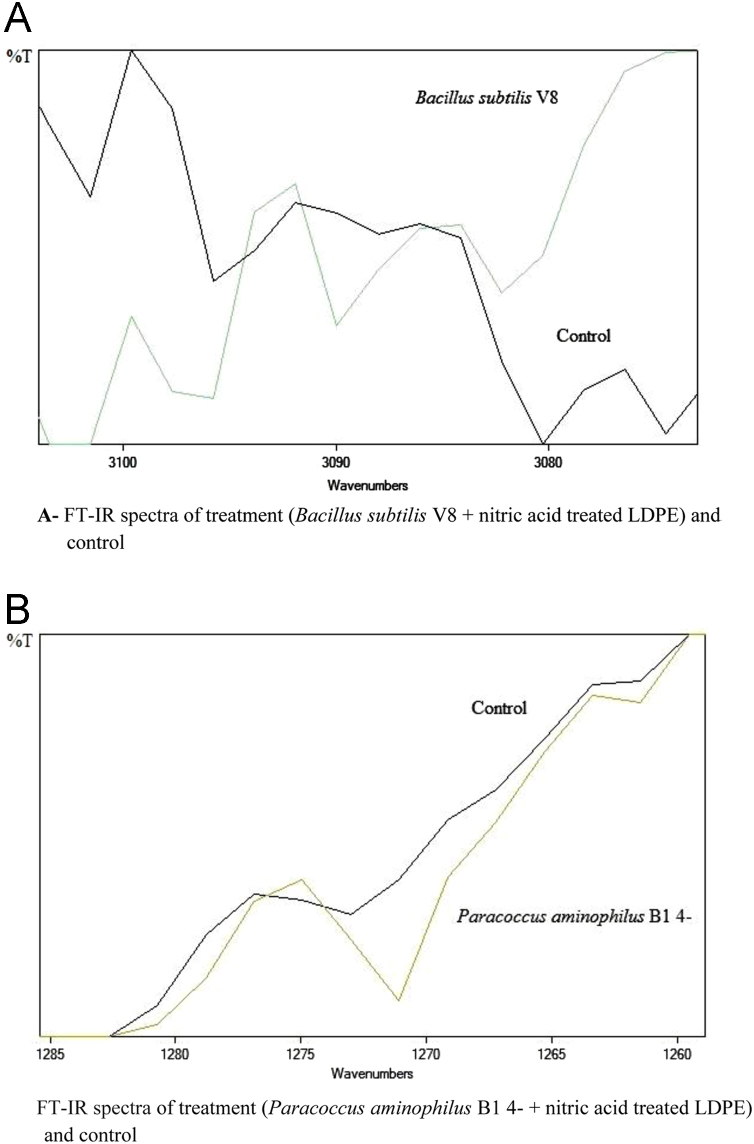

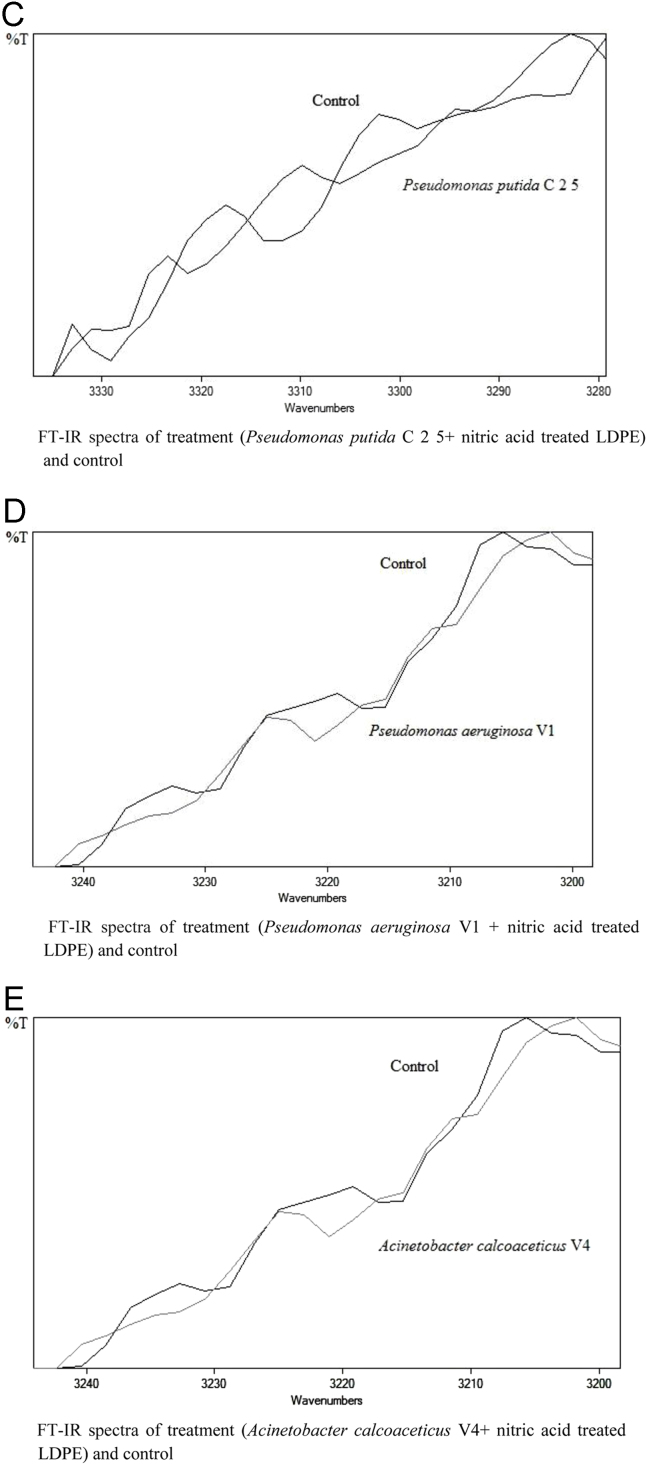
Graph A-E for bacterial treatments with control (untreated LDPE). FT-IR spectra in A for treatment (*Bacillus subtilis* V8 + nitric acid treated LDPE), B for treatment (*Paracoccus aminophilus* B1 4- + nitric acid treated LDPE), C for treatment (*Pseudomonas putida* C 2 5 + nitric acid treated LDPE), D for treatment (*Pseudomonas aeruginosa* V1 + nitric acid treated LDPE) and E for treatment (*Acinetobacter calcoaceticus* V4 + nitric acid treated LDPE).

**Fig. 6:**
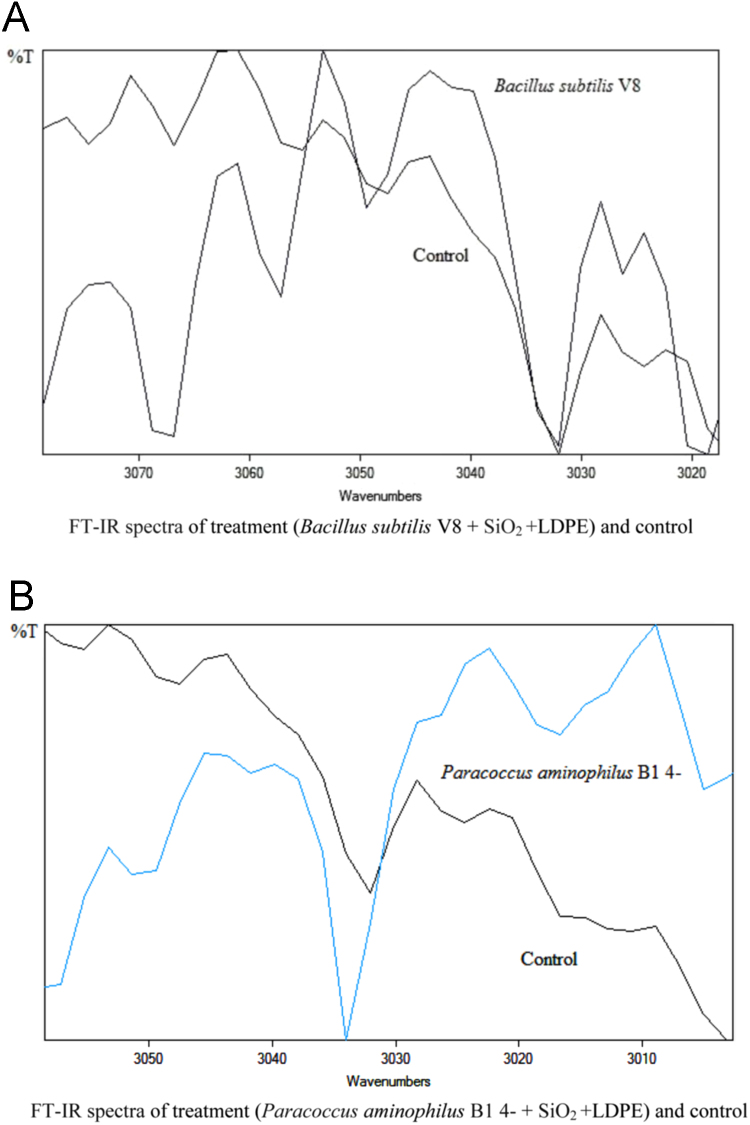

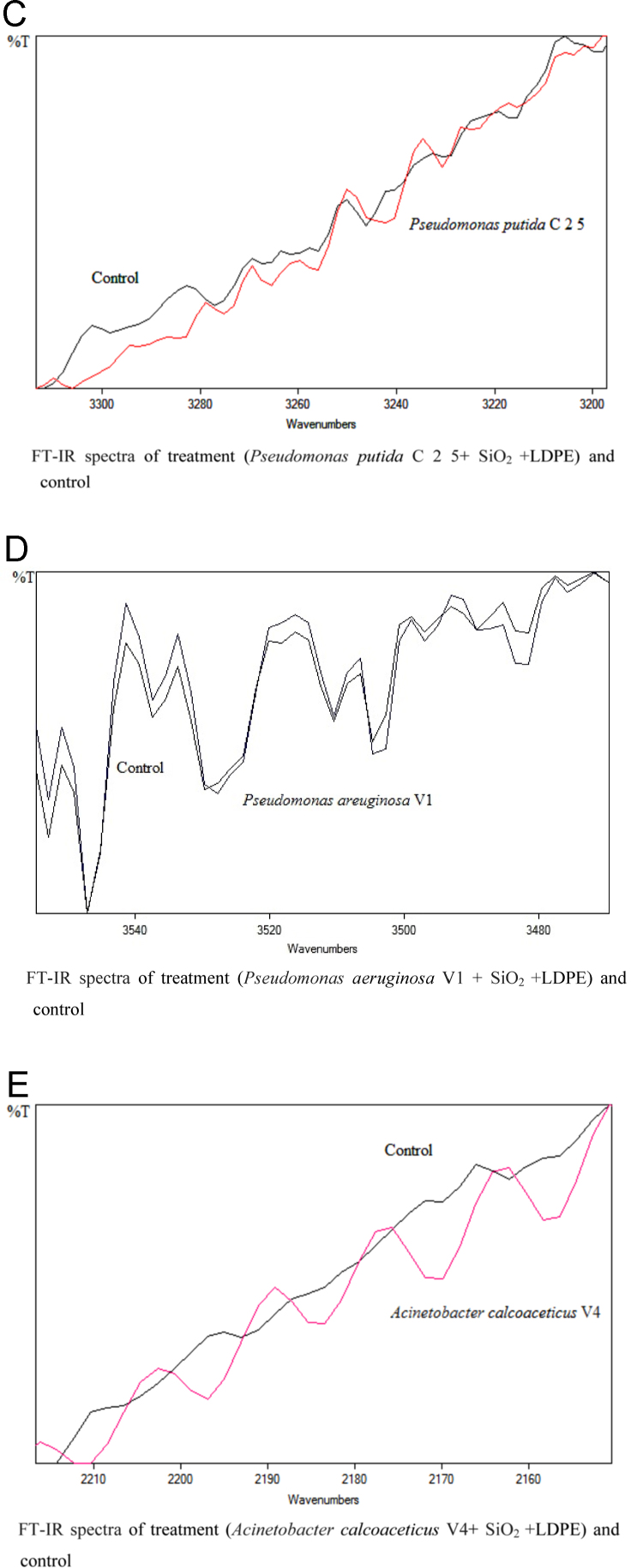
Graph A-E for bacterial treatments with control (untreated LDPE). FT-IR spectra in A for treatment (*Bacillus subtilis* V8 + SiO_2_ + LDPE), B for treatment (*Paracoccus aminophilus* B1 4- + SiO_2_ + LDPE), C for treatment (*Pseudomonas putida* C 2 5 + SiO_2_ + LDPE), D for treatment (*Pseudomonas aeruginosa* V1 + SiO_2_ + LDPE) and E for treatment (*Acinetobacter calcoaceticus* V4 + SiO_2_ + LDPE).

**Fig. 7:**
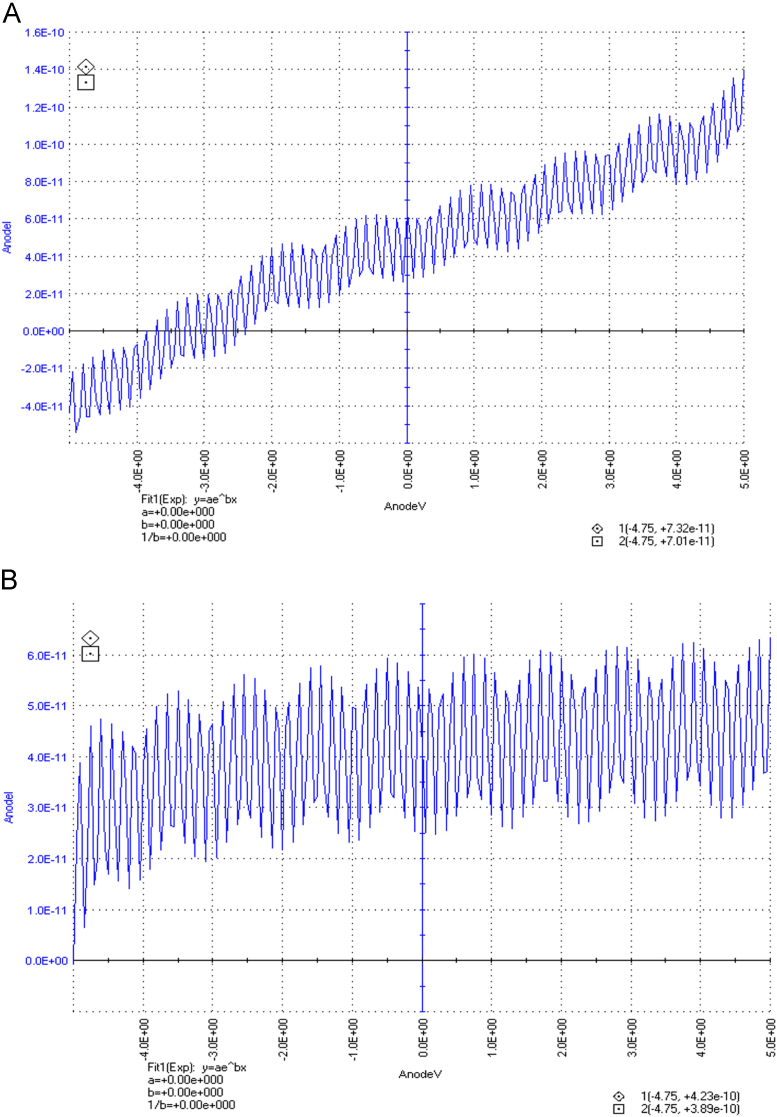

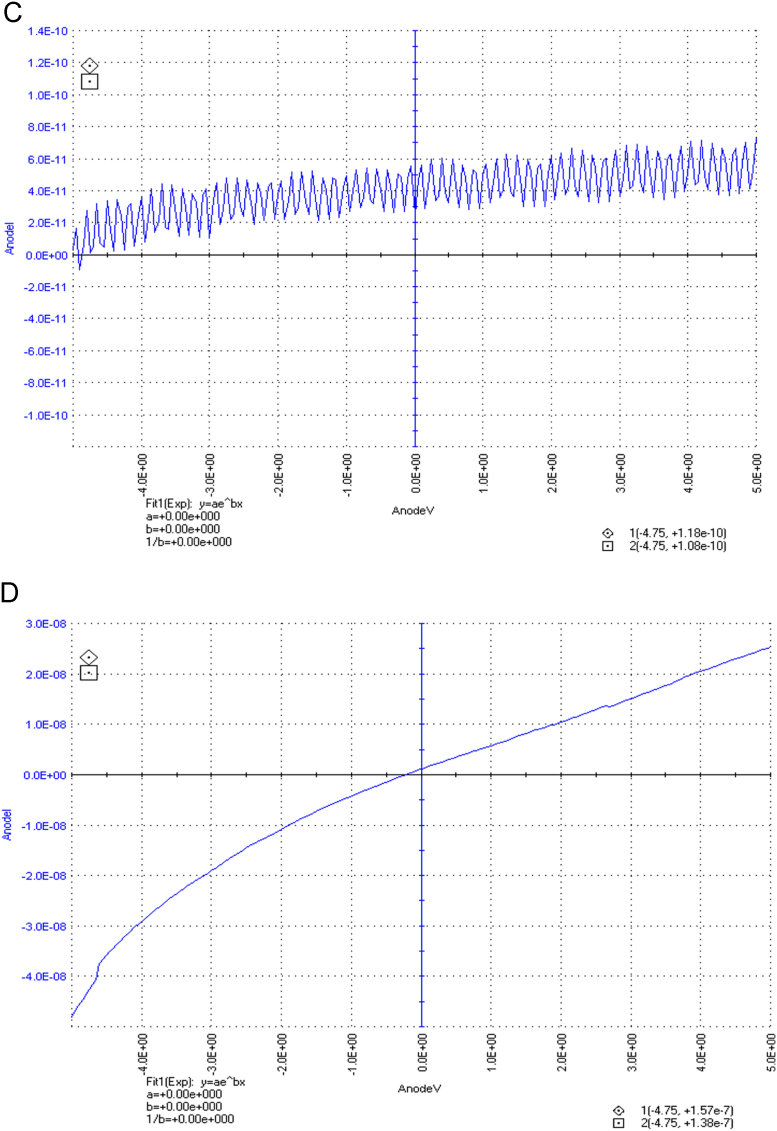

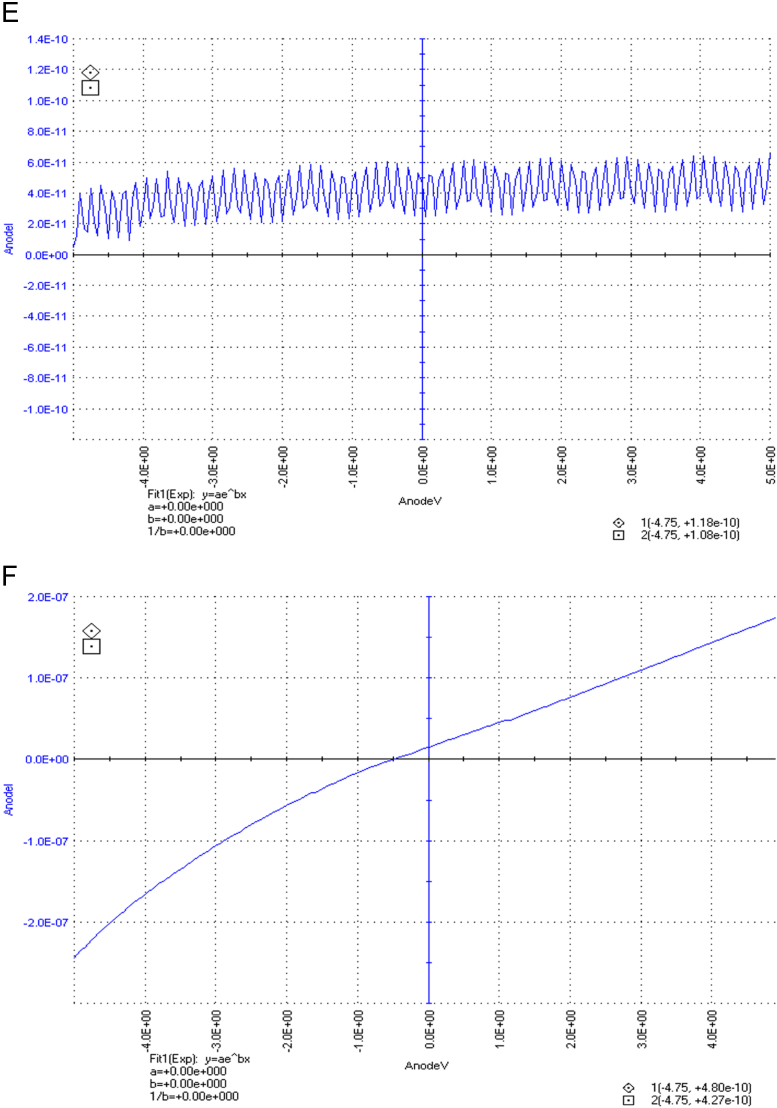
Electrical properties of UV treated biodegraded polymer samples, graph A for control sample (without treatment), B-*Bacillus subtilis* V8+ UV treated LDPE, C-*Paracoccus aminophilus* B1 4-+ UV treated LDPE, D-*Pseudomonas putida* C 2 5+ UV treated LDPE, E- *Pseudomonas aeruginosa* V1+ UV treated LDPE and F-*Acinetobacter calcoaceticus* V4+ UV treated LDPE polymer samples.

**Fig. 8:**
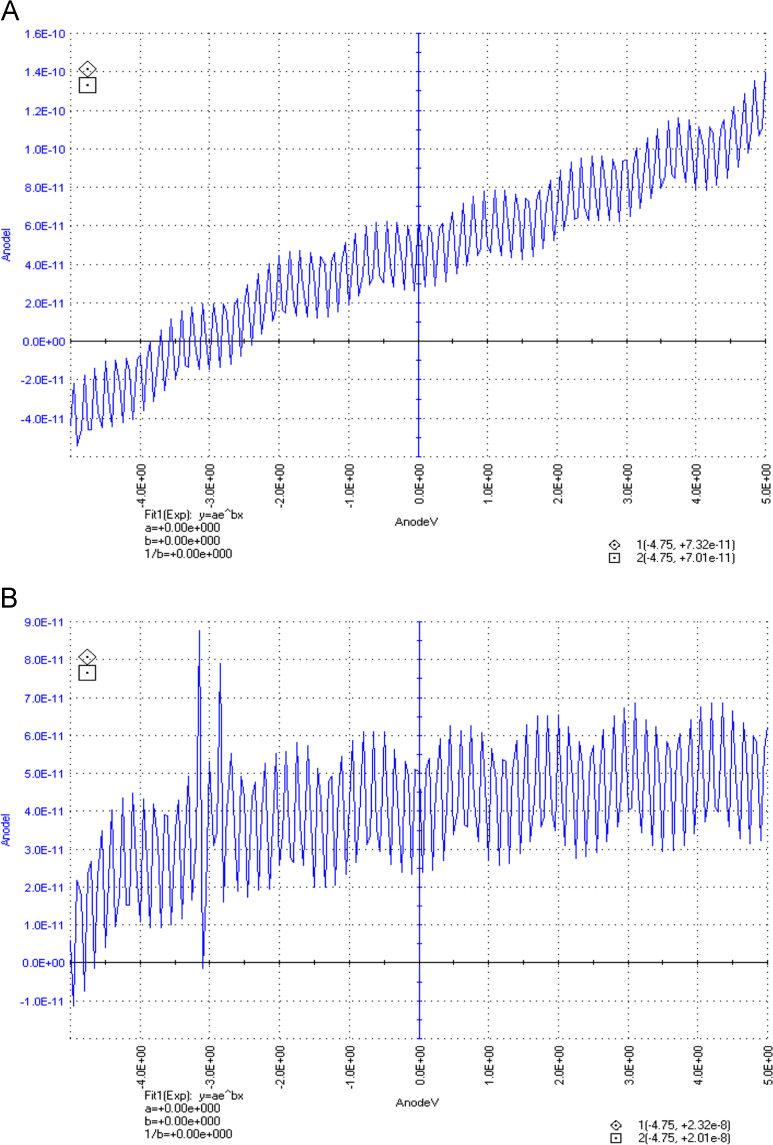

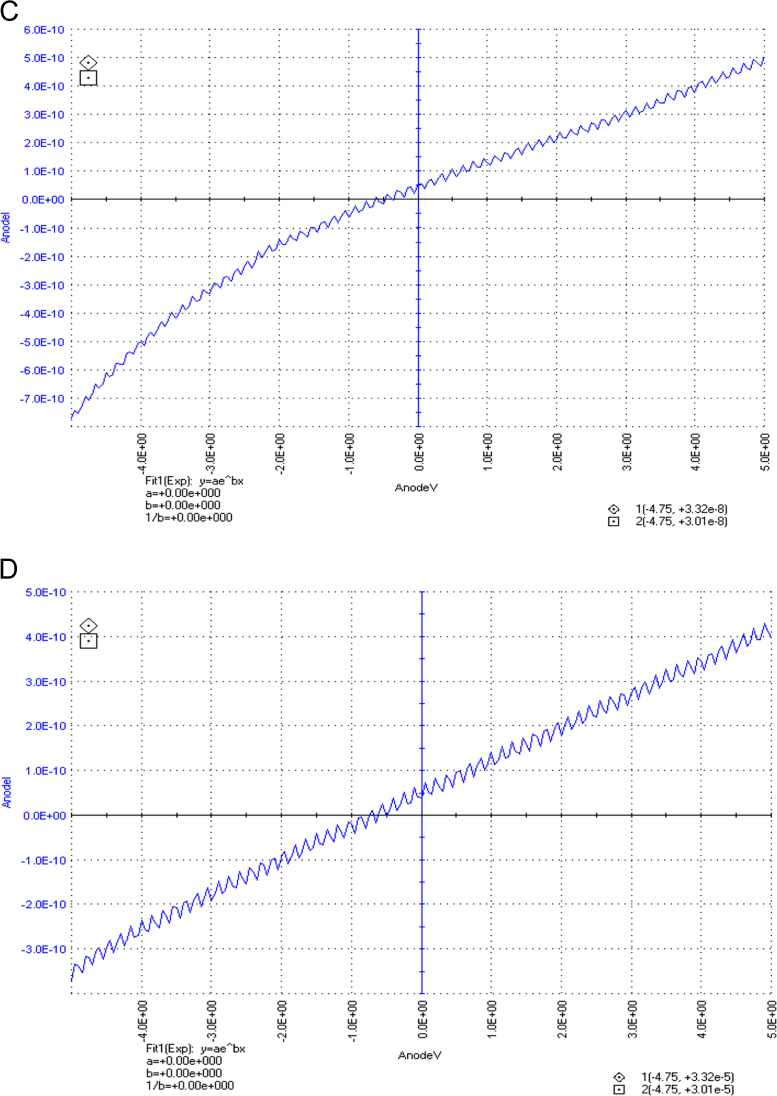

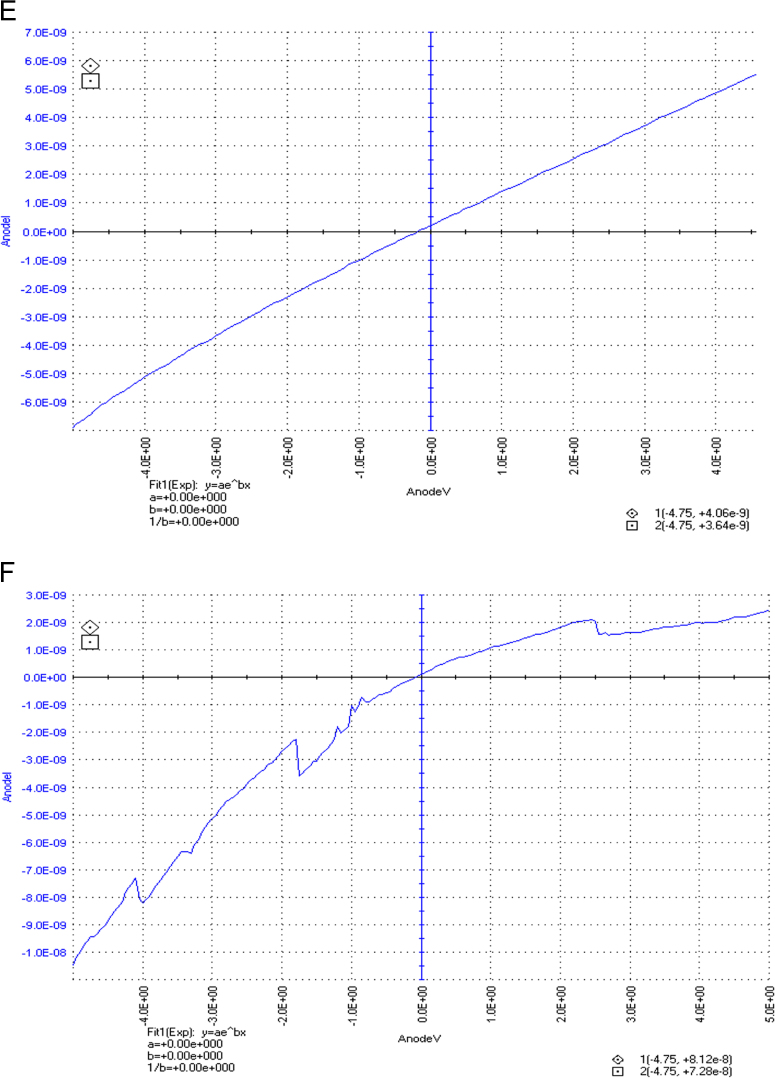
Electrical properties of nitric acid treated biodegraded polymer samples, graph A for control sample (without treatment), B-*Bacillus subtilis* V8+ nitric acid treated LDPE, C-*Paracoccus aminophilus* B1 4-+ nitric acid treated LDPE, D-*Pseudomonas putida* C 2 5+ nitric acid treated LDPE, E- *Pseudomonas aeruginosa* V1+ nitric acid treated LDPE and F-*Acinetobacter calcoaceticus* V4+ nitric acid treated LDPE polymer samples.

**Fig. 9:**
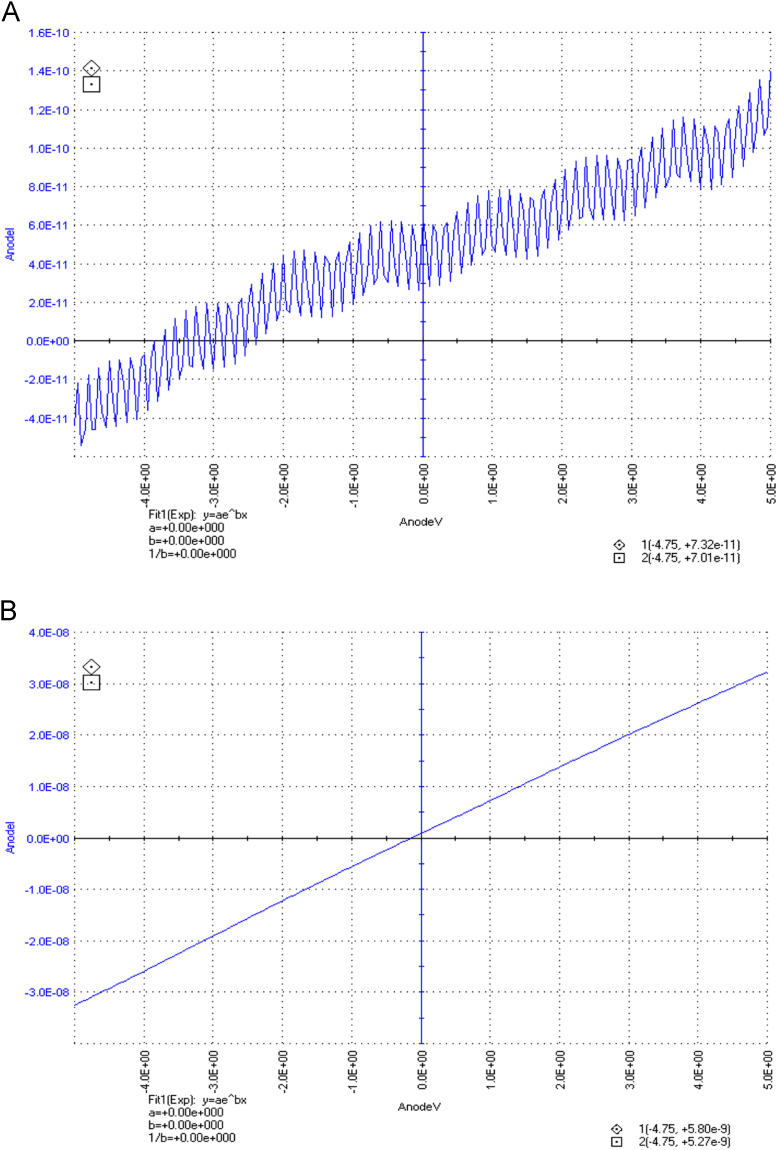

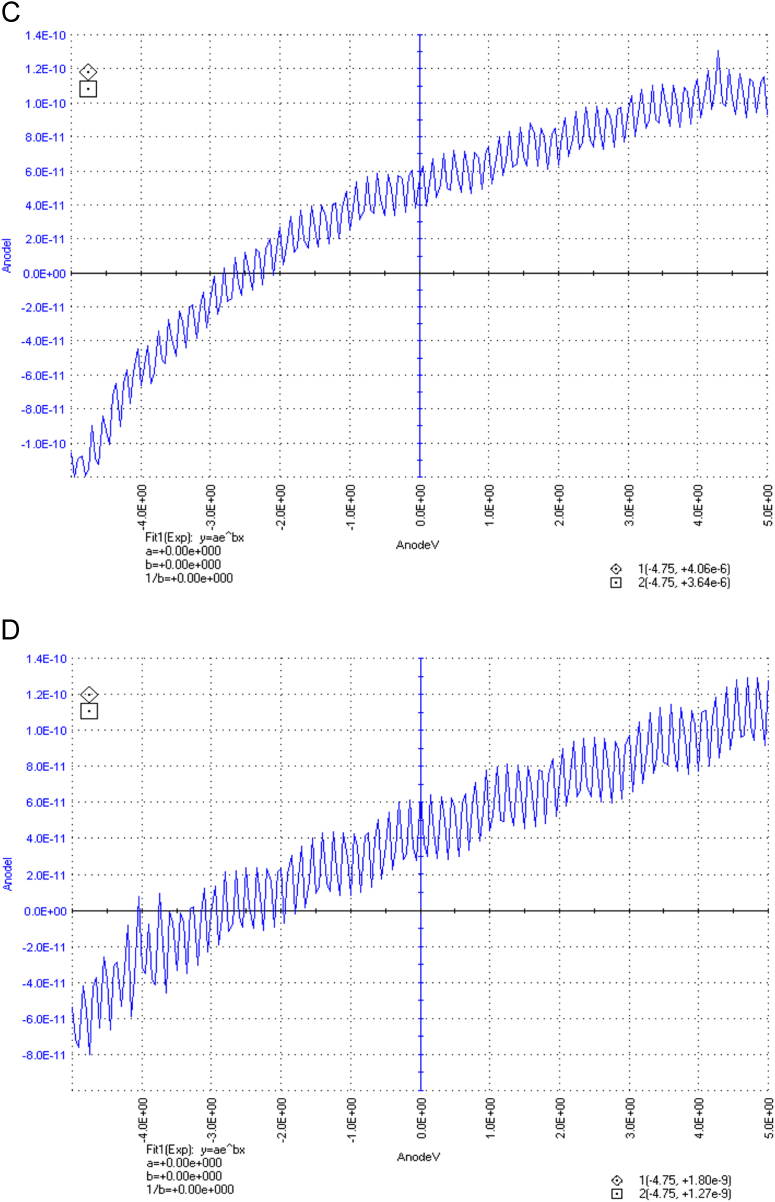

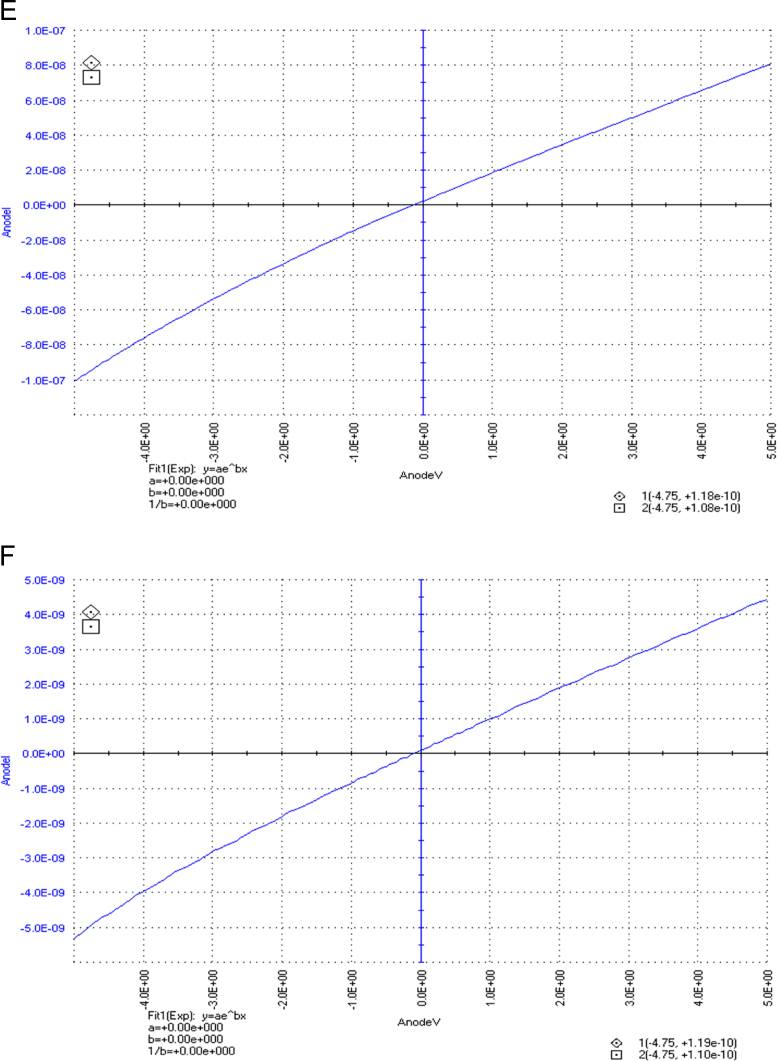
Electrical properties of surfactant treated biodegraded polymer samples, graph A for control sample (without treatment), B-*Bacillus subtilis* V8+ surfactant treated LDPE, C-*Paracoccus aminophilus* B1 4-+ surfactant treated LDPE, D-*Pseudomonas putida* C 2 5+ surfactant treated LDPE, E- *Pseudomonas aeruginosa* V1+ surfactant treated LDPE and F-*Acinetobacter calcoaceticus* V4+ surfactant treated LDPE polymer samples.

The dataset of this article described the consequence of physical and chemical treatments, which include UV, nitric acid and surfactant treatments in LDPE degradation in addition to polymer degrading bacterial strains (*Bacillus subtilis* V8, *Paracoccus aminophilus* B1 4-, *Pseudomonas putida* C 2 5, *Pseudomonas aeruginosa* V1 and *Acinetobacter calcoaceticus* V4). The Fig. 1, Fig. 2, Fig. 3 show the scanning electron microscopy (SEM) micrographs of UV, nitric acid and surfactant treated biodegraded polymer samples, the Fig. 4, Fig. 5, Fig. 6 show the FT-IR characteristics of UV, nitric acid and surfactant treated biodegraded polymer samples with control. Fig. 7, Fig. 8, Fig. 9 show the electrical conductivity property of UV, nitric acid and surfactant treated biodegraded polymer samples. Electrical conductivity examination is an advanced approach for analyzing the electrical properties of polymer. The examination of electrical conductivity is used for determining the consequences for bacterial growth on polymer surface [3], [4], [12], [17], [22]. Bacterial treatment helps in developing electrical conductivity due to bacterial growth on polymer surface and the bacterial presences on it. Such techniques serve as the promising way to analyze the alteration in pure and treated polymer samples. These techniques are facilitated to examine the reproducibility of degradations.

## 2 Experimental design, materials and methods

The consequences of UV, nitric acid and surfactant treatment on polymer degradation in addition to bacterial treatment were determined by SEM, FT-IR and electrical conductivity analysis. Treated polymer samples were exploited in biodegradation experiment to increase the biodegradation ability of isolated bacterial strains. The 365 nm wave length UV light was used to irradiate polymer strips. Polymer (LDPE) strips of 2 cm diameter (weight 0.247–0.408 g) were placed into a UV box (14 by 26 cm) at a distance of 3 cm from the light source for 8 weeks. UV treated polymer strips were aseptically transferred to mineral broth medium and subjected to biodegradation by isolated bacterial cultures [10], [13], [14]. In nitric acid treatment strips were treated with nitric acid (99.0%) at 80 °C for 6 days. After nitric acid treatment polymer strips were aseptically transferred to mineral broth medium and subjected to biodegradation by isolated bacterial cultures [2], [7], [14], [21]. The growth of microorganisms effected in the presence of surfactant. Tween 80 (nonionic surfactant) was added at concentration of 0.05% v/v to the media to test the effect of these substances on bacterial attachment to polymer and LDPE degradation by bacterial isolates compared with control sample [1], [6], [7], [9], [19], [20]. At the end of incubation samples were recovered from culture media and analyzed for degradation by using SEM, FT-IR and electrical conductivity examination [3], [4], [5], [8], [11], [12], [15], [16], [18].

## References

[1] Brandl M.T., Huynh S. (2014). Effect of the surfactant tween 80 on the detachment and dispersal of Salmonella enterica serovar thompson single cells and aggregates from cilantro leaves as revealed by image analysis. Appl. Environ. Microbiol..

[2] Brown B.S., Mills J., Hulse J.M. (1974). Chemical and biological degradation of waste plastics. Nature.

[3] Chang W.Y., Lin C.A., He J.H., Wu T.B. (2010). Resistive switching behaviors of ZnO nanorod layers. Appl. Phys. Lett..

[4] Eswar K.A., Husairi F.S., Mohamad S.A., Azlinda A., Rusop M., Abdullah S. (2013). Synthesis and temperature dependence of IV characteristic of spin-coated nanostructured ZnO on P-type silicon. IOP Conf. Ser. Mater. Sci. Eng..

[5] Gajendiran A., Krishnamoorthy S., Abraham J. (2016). Microbial degradation of low-density polyethylene (LDPE) by *Aspergillus clavatus* strain JASK1 isolated from landill soil. 3 Biotech.

[6] Goto M., Bae H., Lee S.S., Yahaya M.S., Karita S., Wanjae K., Cheng K.J. (2003). Effects of surfactant Tween 80 on forage degradability and microbial growth on the in vitro rumen mixed and pure cultures. Asian Australas. J. Anim. Sci..

[7] Hadad D., Geresh S., Sivan A. (2005). Biodegradation of polyethylene by the thermophilic bacterium Brevibacillus borstelensis. J. Appl. Microbiol.

[8] Jailawi M.H.A., Ameen R.S., Saraf A.A.A. (2015). Polyethylene degradation by *Pseudomonas putida* S3A. Int. J. Adv. Res. Biol. Sci..

[9] Jayashree R., Vasudevan N. (2007). Effect of tween 80 added to the soil on the degradation of endosulfan by *Pseudomonas aeruginosa*. Int. J. Environ. Sci. Technol..

[10] Johnson K.E., Pometto A.L., Nikolov Z.L. (1993). Degradation of degradable starch-polyethylene plastics in a compost environment. Appl. Environ. Microbiol..

[11] Jonsson L.J., Martín C. (2016). Pretreatment of lignocellulose: formation of inhibitory by-products and strategies for minimizing their effects. Bioresour. Technol..

[12] Khanam P.N., AlMaadeed M.A.A. (2015). Processing and characterization of polyethylene-based composites. Adv. Manuf. Polym. Compos. Sci..

[13] Lee B., Pometto A.L., Fratzke A., Bailey T.B. (1991). Biodegradation of degradable plastic polyethylene by Phanerochaete and Streptomyces species. Appl. Environ. Microbiol..

[14] Mahalakshmi V., Siddiq A., Andrew S.N. (2012). Analysis of polyethylene degrading potentials of microorganisms isolated from compost soil. Int. J. Pharma Biol. Arch..

[15] Mehmood C.T., Qazi I.A., Hashmi I., Bhargava S., Deepa S. (2016). Biodegradation of low density polyethylene (LDPE) modiied with dye sensitized titania and starch blend using Stenotrophomonas pavanii. Int. Biodeterior Biodegrad..

[16] Muenmee S., Chiemchaisri W., Chiemchaisri C. (2016). Enhancement of biodegradation of plastic wastes via methane oxidation in semi-aerobic landfill. Int. Biodeterior. Biodegrad..

[17] Sabet M., Soleimani H. (2014). Mechanical and electrical properties of low density polyethylene filled with carbon nanotubes. IOP Conf. Ser. Mat. Sci. Eng..

[18] Sen S.K., Raut S. (2015). Microbial degradation of low density polyethylene (LDPE): a review. J. Environ. Chem. Eng..

[19] Tribedi P., Sil A.K. (2013). Bioaugmentation of polyethylene succinate-contaminated soil with Pseudomonas sp. AKS2 results in increased microbial activity and better polymer degradation. Environ. Sci. Pollut. Res..

[20] Tribedi P., Sil A.K. (2013). Low-density polyethylene degradation by Pseudomonas sp. AKS2 biofilm. Environ. Sci. Pollut. Res..

[21] Yamada-Onodera K., Mukumoto H., Katsuyaya Y., Saiganji A., Tani Y. (2001). Degradation of polyethylene by a fungus, Penicillium simplicissimum YK. Polym. Degrad. Stab..

[22] Yusof Y.M., Shukur M.F., Illias H.A., Kadir M.F.Z. (2014). Conductivity and electrical properties of corn starch–chitosan blend biopolymer electrolyte incorporated with ammonium iodide. Phys. Scr..

